# Retrospective epidemiological study in a reference service for rare diseases, Salvador, 2018-2019

**DOI:** 10.1590/S2237-96222026v35e20240832.en

**Published:** 2026-04-11

**Authors:** Camila Ferreira Ramos, Ney Cristian Amaral Boa Sorte, Angelina Xavier Acosta

**Affiliations:** 1 Universidade Federal da Bahia, Programa de Pós-Graduação em Medicina e Saúde, Salvador, Bahia, Brazil Universidade Federal da Bahia Programa de Pós-Graduação em Medicina e Saúde Salvador Bahia Brazil; 2 Hospital Universitário Professor Edgard Santos, Núcleo de Avaliação em Tecnologias em Saúde, Salvador, BA, Brazil. Hospital Universitário Professor Edgard Santos Núcleo de Avaliação em Tecnologias em Saúde Salvador BA Brazil; 3 Universidade Federal da Bahia, Departamento de pediatria da Faculdade de Medicina da Bahia, Salvador, BA, Brazil. Universidade Federal da Bahia Departamento de pediatria da Faculdade de Medicina da Bahia Salvador BA Brazil

**Keywords:** Health Surveys, Rare Diseases, Genetics, Health Services, Hospitals, University, Encuestas Epidemiológicas, Enfermedades Raras, Genética, Servicios de Salud, Hospitales Universitarios

## Abstract

**Objective:**

To describe an epidemiological survey involving individuals with rare diseases who attended from 2018 to 2019 in a reference service for rare diseases in Bahia.

**Methods:**

The data were obtained from medical records, using three forms named "identification," "diagnosis," and "treatment," which were subsequently entered into the REDCap software and analyzed descriptively.

**Results:**

A total of 1,744 participants were included, of whom 69.3% were in the 0-19 age group (mean age of 15 years), with a predominance of female individuals (51.1%). Most cases had a confirmed diagnosis, while cases with a suspected diagnosis and undefined diagnosis accounted for 20.1% and 18.0%, respectively. Most diagnoses were made clinically, 58.4%. The mean age of onset of symptoms was 7.5 years. Family recurrence was reported in 36.7% of cases, and consanguinity in 16.1%. The mean maternal age at conception was 26.6 years, and the paternal age was 31.0 years. Approximately 94.0% of rare diseases were of genetic origin, of which 69.4% were included in the international disease classifications of the National Policy for the Comprehensive Care of Persons with Rare Diseases (*Política Nacional de Atenção Integral às Pessoas com Doenças Raras*, PNAIPDR).

**Conclusion:**

A predominantly young sample was observed, with most having a confirmed diagnosis but experiencing delays in diagnosis, thus characterizing the sociodemographic and clinical profile of rare diseases treated at the service, whose information has been relevant for planning strategies regarding surveillance and the provision of care for this population.

## Resumen

**Objetivo:**

Describir una encuesta epidemiológica que involucró a individuos con enfermedades raras atendidos durante el período 2018-2019 en un servicio de referencia en enfermedades raras de Bahía.

**Métodos:**

Los datos se obtuvieron a partir de los historiales clínicos, utilizando tres formularios denominados "identificación", "diagnóstico" y "tratamiento", posteriormente ingresados en el software REDCap y analizados de forma descriptiva.

**Resultados:**

Se incluyeron 1.744 participantes, de los cuales el 69,3% pertenecía a los grupos de edad de 0-19 años (mediana de 15 años), predominando el sexo femenino (51,1%). La mayoría de los casos tenía diagnóstico confirmado, mientras que los casos con diagnóstico sospechoso y sin diagnóstico definido representaban el 20,1% y 18,0%, respectivamente. La mayoría de los diagnósticos se realizó clínicamente (58,4%). La edad promedio al inicio de los síntomas fue de 7,5 años. La recurrencia familiar se reportó en el 36,7% de los casos y la consanguinidad en el 16,1%. La edad materna promedio en la concepción fue de 26,6 años y la edad paterna de 31,0 años. Alrededor del 94,0% de las enfermedades raras fueron de origen genético, de las cuales el 69,4% estaban incluidas en las clasificaciones internacionales de enfermedades de la Política Nacional de Atención Integral a las Enfermedades Raras (*Política Nacional de Atenção Integral às Pessoas com Doenças Raras*, PNAIPDR).

**Conclusión:**

Se observó una muestra predominantemente joven, la mayoría con diagnóstico confirmado, aunque con retraso en la confirmación diagnóstica, caracterizando así el perfil sociodemográfico y clínico de las enfermedades raras atendidas en el centro, cuyas informaciones resultan relevantes para la planificación de estrategias de vigilancia y provisión de cuidados a esta población.

**Ethical aspects**

This research respected ethical principles, having obtained the following approval data:

Research ethics committee: Universidade Federal da Bahia

Opinion number: 5,961,780

Approval date: 24/3/2023

Certificate of submission for ethical appraisal: 33970820,0,3001,0049

Informed consent record: Exempt.

## Introduction 

Rare disease is the term used to describe conditions that are generally chronic and debilitating, with low individual incidence and/or prevalence ^1^. Countries such as Australia, the United States, Japan, and the United Kingdom have a prevalence of 1.1; 7.5; 4.0; and 0.18 per 10,000 inhabitants, respectively ^2^. In Brazil, the Ministry of Health defines rare disease according to the World Health Organization criterion, that is, 1.3 per 2,000 individuals or 65 per 100,000 individuals ^3^. Although individually rare, collectively they affect approximately 10.0% of the population. It is estimated that there are around 6,000 conditions, 80.0% of which are of genetic origin ^4^, with a worldwide prevalence of approximately 3.5% to 5.9%, that is, about 260,000,000 to 440,000,000 individuals. According to these estimates, between 7,000,000 and 16,000,000 individuals in Brazil are affected by rare diseases, posing a significant public health challenge ^5^. 

Brazilian epidemiological data on these diseases are still scarce, and many are based on international registries, especially sponsored by the pharmaceutical industry and addressing specific disease groups. This aspect restricts the knowledge of the data for managers and policymakers. However, some important advances occurred, with emphasis on the creation of the National Policy for Comprehensive Care for People with Rare Diseases (*Política Nacional de Atenção Integral às Pessoas com Doenças Raras*, PNAIPDR) in the Brazilian National Health System (*Sistema Único de Saúde*, SUS), established by Ordinance No. 199, of 30/1/2014 ^3^. This policy aims to help reduce morbidity and mortality by improving the quality of life of individuals through promotion, prevention, early detection, treatment, and palliative care. In this policy, rare diseases are classified into two major groups: those of genetic etiology, including congenital anomalies, intellectual disability, and inborn errors of metabolism; and those of the group of non-genetic diseases (infectious, immunological, and autoimmune) ^3^. 

Considering that the National Policy for Comprehensive Care for People with Rare Diseases (PNAIPDR) under the Brazilian National Health System (SUS) did not link the diagnostic and care process to a structured data registry-beyond the completion of the authorization for high-complexity procedures-the establishment of the National Network of Rare Diseases (*Rede Nacional de Doenças Raras*, RARAS) project, which is conducting a national census on rare diseases with the description of clinical, sociodemographic, and therapeutic pathway aspects across 34 health services in Brazil, constitutes a major initiative to provide data for public health management. In Bahia, the fourth most populous state in Brazil ^4^, and based on the aforementioned estimates, between 525,000 and 885,000 individuals are estimated to be living with rare diseases.

In this context, this study aims to describe the findings of the National Rare Diseases Network (RARAS) census during its first two years of implementation within the largest reference center for rare diseases in Bahia.

## Methods 

Study design and context

This is a multicenter observational study with a cross-sectional design, using data collected in 2022 and 2023 from cases of rare diseases attended in 2018 and 2019 at the genetics and pediatric nutrition and nutrology outpatient clinics of the Professor Edgard Santos University Hospital (*Hospital Universitário Professor Edgard Santos*), Federal University of Bahia (*Universidade Federal da Bahia*, UFBA). 

Bahia has an area of 564,760,429 km², with a population of 14,141,626 inhabitants. For health management purposes, the state is divided into nine Regional Health Centers, with the focus specifically on the Eastern office, which includes the state capital, Salvador. 

Participants/study size

All cases attended at the center in 2018 and 2019 with a confirmed diagnosis, suspected diagnosis, or undefined diagnosis of a rare disease were included, with a waiver of the requirement for the informed consent form and assent form, given the retrospective nature of the data collected. Incomplete data were not exclusion criteria. The included cases were obtained from a search of records in the hospital's electronic and paper medical records. 

Data sources

For data collection using the REDCap application, three forms were used: "identification," containing sociodemographic data; "diagnosis," containing information on the form and characterization of the diagnosis; and "treatment," containing data related to the therapeutic management of the cases. 

Bias

The team responsible for data collection was previously trained by the center's lead researchers in order to minimize the risk of information bias.

Variables

Variables collected included race/skin color, region of residence and birth, sex, age, rare disease diagnostic status (confirmed/suspected/undefined), type of diagnosis (etiological or clinical), consanguinity (yes/no), family recurrence (yes/no), timing of diagnosis (prenatal, neonatal, or postnatal), funding source for the diagnosis, age at symptom onset, whether specific treatment for the rare disease was provided, type of treatment, funding source for the treatment, and follow-up with another specialty. 

Statistical methods

Data were presented using descriptive statistics, with simple frequencies for categorical variables and measures of central tendency and dispersion for quantitative variables. The prevalences of the most frequently identified diseases were calculated as the ratio between the total number of cases and the average population of Bahia, estimated for 2018 and 2019, and expressed per 1,000,000 inhabitants. The data were analyzed using Microsoft Excel, Office 365 version.

## Results 

A total of 1,744 participants were included in the study, of whom 51.1% were female and 76.3% self-identified as Brown (Brazilian mixed race). Most of these individuals were born in the Northeast region of Brazil (97.2%), with the largest proportion (43.4%) from the Eastern Regional Health Center of Bahia, where the study center is located. The state capital, Salvador, had the highest frequency of cases (34.0%). Regarding participants' age, the majority (69.3%) were between 0 and 19 years, with a median of 15.0 (8.0-25.0) years (Table 1). 

The regional health center with the highest proportion of participants was the Eastern Center (48.1%), with Salvador accounting for 71.8% of the cases, followed by the Central East, South, Central North, Southwest, Northeast, North, West, and Far South Centers (20.6%; 7.4%; 6.2%; 5.6%; 4.5%; 4.4%; 2.4%; 0.8%, respectively). 

Regarding diagnostic status, 1,084 participants (61.9%) had a confirmed diagnosis, 352 participants (20.1%) had a suspected diagnosis, and 315 participants (18.0%) had an undefined rare disease diagnosis (Figure 1). Most diagnoses were based on clinical criteria (58.4%), and among the cases with an etiological diagnosis (41.6%), the biochemical method accounted for the highest proportion (34.0%), followed by cytogenetic (32.4%), molecular (17.9%), and histopathology methods (34.0%). In addition, seven individuals had more than one diagnosis (1,751 records from 1,744 individuals). 

In the majority of cases, the diagnosis occurred during the postnatal period (97.0%). The mean age at symptom onset was 7.5 (±12.8) years, and the median (p25-p75) was 2.0 (1.0-7.0) years. Family recurrence was reported in 36.7% of cases, and consanguinity was observed in 16.1% of cases. Regarding maternal age at the birth of the individual with a rare disease, the mean (SD) was 26.6 (6.8) years, ranging from 13 to 47 years. Paternal age, in turn, showed a mean (SD) of 31.0 (8.3) years, ranging from 14 to 65 years, as detailed in Table 2.

**Table 1:** Sociodemographic characteristics of the retrospective epidemiological study. Salvador, 2018-2019 (n=1,744)

Variables	n (%)
Sex	
Male	851 (48.8)
Female	892 (51.1)
Undefined	1 (0.1)
Region of birth (regional health center) - Bahia	
Southwest	99 (5.7)
South	135 (7.7)
West	44 (2.5)
North	74 (4.2)
Northeast	63 (3.6)
East	755 (43.4)
Far South	14 (0.8)
Central North	99 (5.7)
Central East	290 (16.6)
Not informed	171 (9.8)
Age group (years)	
0-5	478 (27.4)
6-19	731 (41.9)
20-59	487 (27.9)
≥60	48 (2.8)

**Figure 1:**
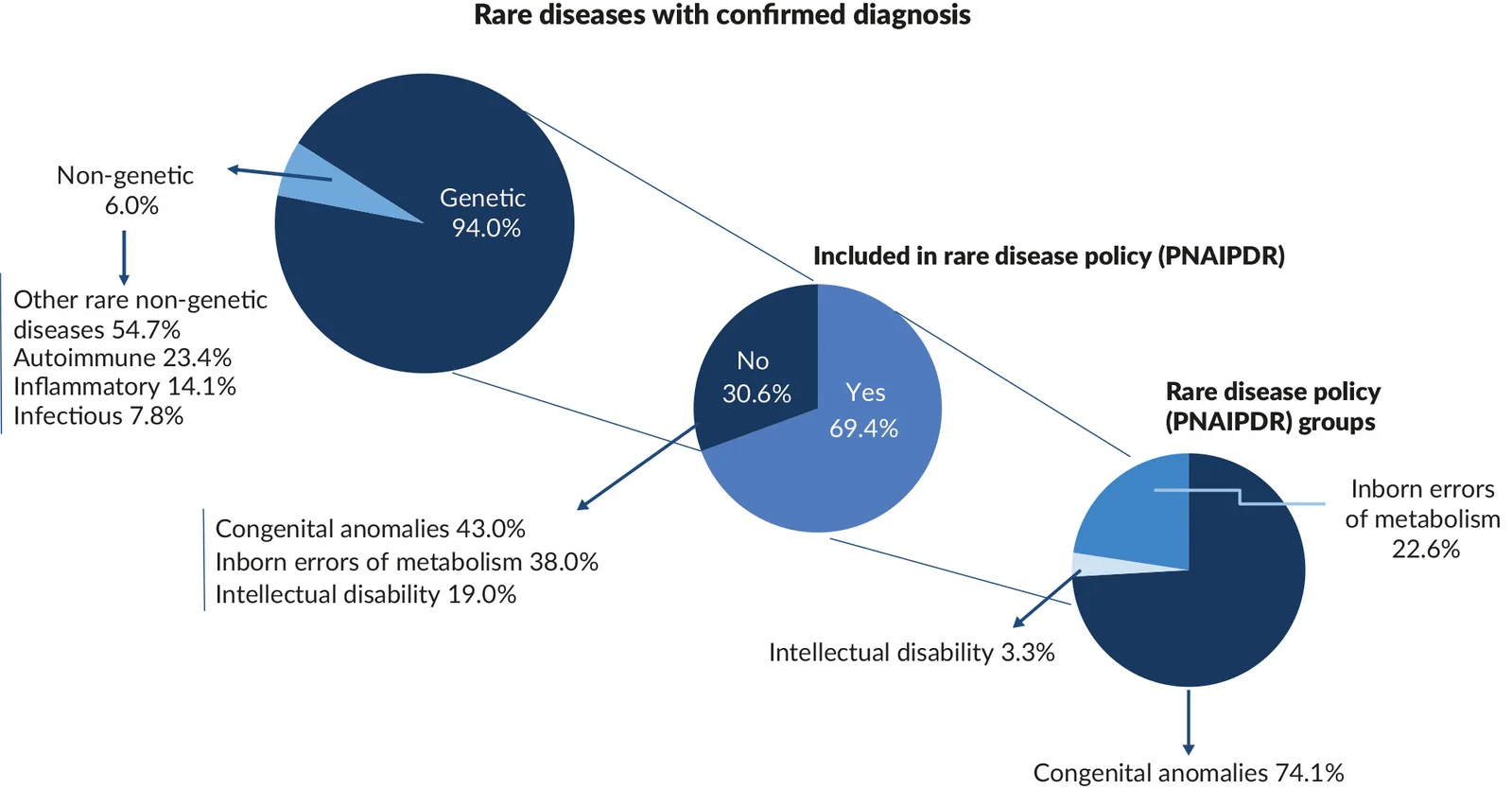
Diagnostic status of the retrospective epidemiological study. Salvador, 2018-2019 (n=1,751)

Among the cases with a confirmed diagnosis, the most prevalent diseases at the study center are described in Table 3. A more detailed analysis of the classification of confirmed rare disease diagnoses showed that 94.0% were of genetic origin, of which 69.4% were included in the list of diseases coded by the International Statistical Classification of Diseases (ICD) and covered by the national policy for comprehensive care of people with rare diseases. Among these listed diseases, 74.1% belonged to the group of congenital anomalies, 22.6% to the group of inborn errors of metabolism, and 3.3% were classified in the intellectual disability group. Regarding rare diseases of non-genetic origin, 7.8% were classified as infectious, 14.1% as inflammatory, 23.4% as autoimmune, and 54.7% as other rare diseases of non-genetic origin. 

As not all diagnosed rare diseases were included in the national policy list for comprehensive care of people with rare diseases, among the diseases coded by the International Classification of Diseases but not listed in the policy, 43.0% belonged to the group of congenital anomalies, 38.0% were classified as inborn errors of metabolism, and 19.0% as intellectual disability (Figure 2). 

## Discussion 

Studies on the epidemiology of rare diseases in Brazil are scarce; therefore, this work aimed to contribute comprehensive information on individuals with rare diseases at the referral center for rare diseases of the Professor Edgard Santos University Hospital, to improve understanding of this population in a highly representative center in Bahia. 

**Table 2:** Clinical characteristics of the retrospective epidemiological study. Salvador, 2018-2019 (n=1,751)

Variables	n (%)
Timing of diagnosis	
Prenatal	11 (1.0)
Neonatal screening	22 (2.0)
Postnatal	1,051 (97.0)
Total	1,084 (100.0)
Family recurrence	
Yes	524 (36.7)
No	896 (63.3)
Subtotal	1,420 (100.0)
Not informed	331 (18,9)
Consanguinity	
Yes	193 (16.1)
No	1,002 (83.9)
Subtotal	1,195 (100.0)
Not informed	556 (31.7)

**Figure 2:**
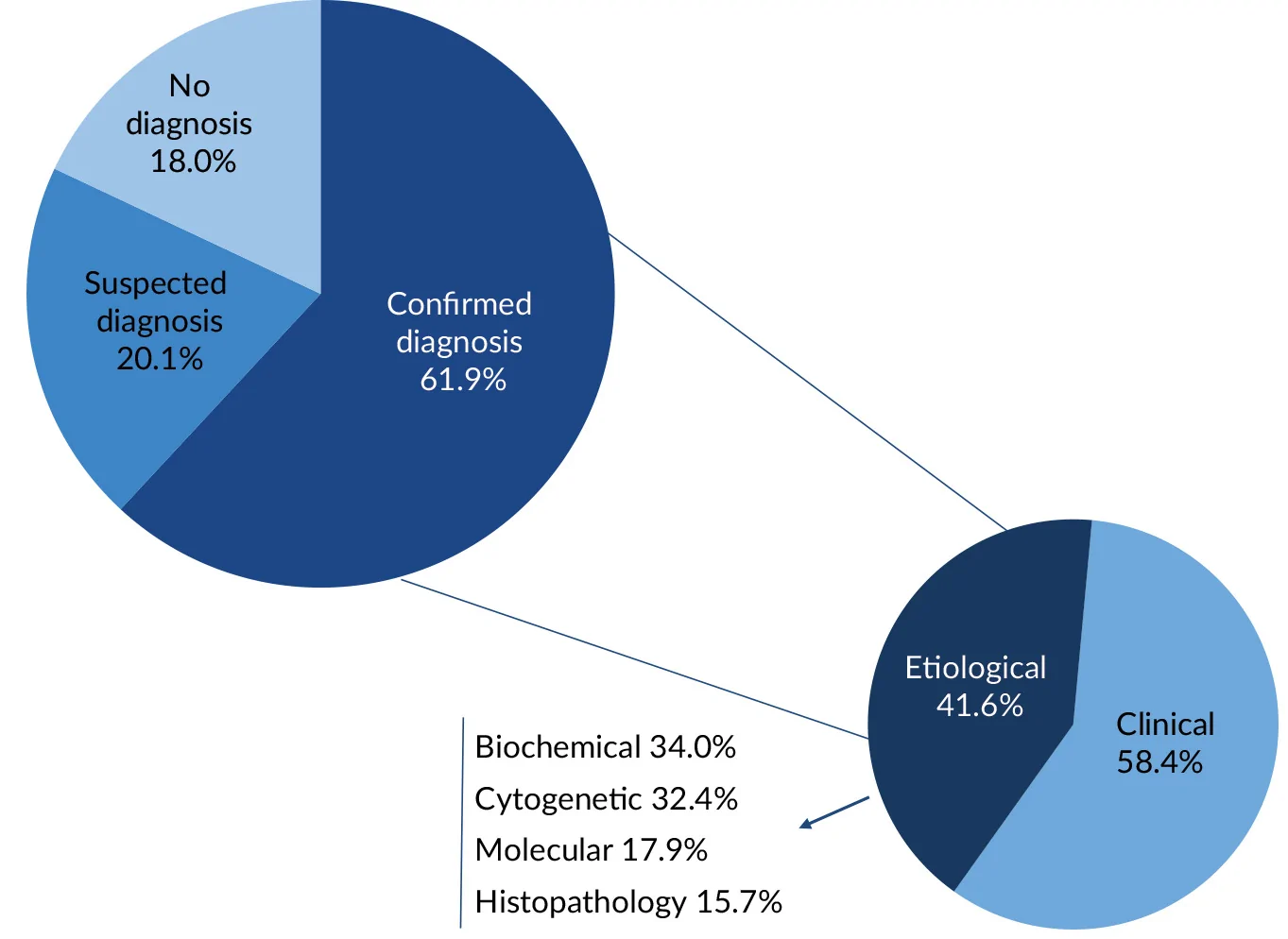
Distribution of rare diseases of genetic and non-genetic origin in the retrospective epidemiological study at Professor Edgard Santos University Hospital. Salvador, 2018-2019 (n=1,084)

Some operational limitations were identified in the present study regarding the data source, since it was based on medical record review. The lack of standardized medical notes resulted in gaps in the information recorded. However, there was no major compromise, as strategies were employed to ensure data completeness through a detailed review of the information available in medical notes, based on a standardized data collection process carried out by a trained team under constant supervision by the research coordination.

The sociodemographic data of this study reflected the latest demographic census of the Brazilian Institute of Geography and Statistics (*Instituto Brasileiro de Geografia e Estatística*, IBGE) for Bahia, in which 51.7% of the population is female, compared to 51.1% of participants in this study. Similar findings were reported in a national-level study, in which 50.6% of the sample was female ^6^. 

Regarding race/skin color, the most prevalent was Brown (66.7%), followed by Black (10.6%). According to the 2022 IBGE census for Bahia, the proportion of individuals self-identified as Brown was lower (57.3%), while the proportion of those self-identified as Black was higher (22.4%) ^7^. The national-level study reported Brown as the most frequent race/skin color (47.4%), followed by White (45.9%), most likely due to the participation of individuals from the South and Southeast regions, where the population is predominantly White ^6^.

The majority of the study sample (61.8%) were in the 0-19 age group, with 20.1% of individuals being up to 6 years old. In one study, 69.9% of the cases were within the pediatric group, corroborating the higher prevalence of rare disease cases in childhood and adolescence, as these are severe conditions with early onset and detection in this age group ^8^.

Regarding the region of residence, Bahia's Eastern Regional Health Center showed the highest prevalence (48.1%) of rare diseases, as it encompasses Salvador, where the study center is located, and which has the largest population in the state ^7^. Considering the data on individuals with rare diseases relative to the total population in each region, 0.017% of residents in the Eastern region were affected, followed by 0.01% in the Central-East region and 0.007% in the Southern region. The lower percentages of participants from regions farther from the capital are likely due to limited access to the center, follow-up at closer facilities (including in other states), or genuinely lower prevalence in those areas, highlighting the need for a better understanding of rare disease prevalence in more remote regions. 

Although most individuals had a confirmed diagnosis (61.9%), a significant proportion still presented with a suspected diagnosis (20.1%) or without a defined diagnosis (18.0%). These high rates of cases without a defined diagnosis can be explained by the limited access to molecular diagnostic techniques at the time of data collection, a situation similar to that observed in other public services in Brazil ^9^. National-level data showed that cases still classified as diagnostic suspicion accounted for 17.0% of the sample ^6^. Considering the complexity of rare diseases, diagnosis can be challenging even in developed countries such as the United States and Australia, which have specialized services and better access to genetic testing, yet still present a significant number of undiagnosed cases ^10^. Prior to the implementation of the policy, national-level studies showed that molecular testing was available in 50.0% of the genetic services listed within the Brazilian National Health System (SUS) ^11^. A study evaluating the availability of genetic tests in public services in Brazil reported similar findings to previous studies, with a presence of 54.05% ^9^.

The majority (58.4%) of cases with a confirmed diagnosis were identified clinically, while 41.6% had a defined etiology, consistent with national-level data. Regarding the type of diagnosis, biochemical methods accounted for the highest percentage (34.0%), similar to the national study, which reported 42.5% ^6^. Regarding molecular diagnosis, the study center showed a diagnostic confirmation rate of 17.9%, compared to 30.9% observed nationally in Brazil. This difference was nearly half, which can be explained by the greater availability of molecular tests in the South and Southeast regions, as these services have better-equipped laboratories and broader access to laboratory exams compared to other Brazilian states ^6^. The greater availability of these tests certainly enables a more accurate diagnosis, thereby reducing the impact of the diagnostic journey ^12^.

The consanguinity rate was 16.1%, markedly higher than the national data (6.4%), consistent with previous studies in the Northeast region of Brazil, which show higher rates of consanguinity and endogamy, ranging from 6.0% to 12.0% ^13^. A study conducted in Rio Grande do Norte showed that municipalities with greater geographic isolation had higher percentages of related individuals (30.0%) compared to areas with higher mobility (9.0%). On the other hand, previous studies reported lower rates of related individuals in South America (0.96%) and in Brazil (1.60%) ^14^. The majority (52.1%) of consanguineous individuals resided in municipalities located in the interior of the state, many in rural areas, where consanguineous marriages are more frequent for various reasons, such as cultural practices and geographic isolation ^13^. 

Another risk factor associated with the occurrence of genetic diseases is parental age at conception. However, the mean maternal and paternal ages observed were 26 and 31 years, respectively, lower than those reported in the literature ^15^^-^^16^. Thus, a priori, this risk factor did not appear to be significant; however, a more detailed analysis is necessary to understand the vulnerability of the studied population and, consequently, to contribute to improved prevention strategies.

Regarding the age at symptom onset, it was 7.5 years, similar to that reported in the national study, which had a mean of 5.4 years ^6^. This delay may be even greater in developing countries, such as Brazil ^17^, where the diagnostic journey can be a determining factor in increasing the financial burden related to diagnosis and/or treatment, as well as in worsening the quality of life for individuals affected by rare diseases and their families ^18^.

Among the diagnosed rare diseases, the ten most prevalent accounted for 39.7% of the studied cases, while the remaining diagnoses (60.3%) corresponded to diseases with very low individual frequency. These results are consistent with the Orphanet database, in which the majority (84.5%) of diseases have very low prevalence, in contrast to a small group of rare diseases with higher prevalence ranges ^18^. It is noted that the ten most prevalent diseases (Table 3) belonged to the group of congenital anomalies, according to the National Policy for Comprehensive Care of People with Rare Diseases (PNAIPDR), with osteogenesis imperfecta being the most prevalent (9.6 per 1,000,000 inhabitants). In Brazil, the prevalence of this disease is still unclear, as data on the total number of diagnosed cases in official platforms are scarce ^19^. It is estimated that most individuals affected by osteogenesis imperfecta in Bahia are followed at the study center, as it is the only public service in the state qualified to treat this disease ^20^. This higher prevalence accounted for 37.8% of the national caseload, where osteogenesis imperfecta ranked as the 4fourth most frequent disease. This result must be interpreted with great caution, as there is a clear selection bias in the data. The analyzed center is a reference for many of the most prevalent rare diseases described, which explains this distribution.

In the present study, 94.0% of rare diseases were of genetic origin (Figure 1), a higher percentage than reported in the literature, where approximately 80.0% of rare diseases are of genetic origin ^21^. Despite the strategies employed to minimize selection bias, as described in the methodology, this percentage was influenced by the fact that most of the cases came from patients followed in genetics outpatient clinics. It is assumed that rare diseases of non-genetic origin are underreported for the methodological reasons already explained. 

**Table 3:** Most prevalent rare diseases in the retrospective epidemiological study. Salvador, 2018-2019 (n=431)

Most prevalent rare diseases	n (%)	Observed prevalence^a^
Osteogenesis imperfecta	136 (31.5)	9.6
Turner syndrome	58 (13.5)	4.1
Muscular dystrophy	56 (13.0)	3.9
Adrenogenital disorders	47 (10.9)	3.1
Mucopolysaccharidosis	40 (9.3)	2.8
Cystic fibrosis	22 (5.1)	1.6
Hereditary angioedema	22 (5.1)	1.6
Epidermolysis bullosa	19 (4.4)	1.3
Glycogen storage disease	16 (3.7)	1.1
Neurofibromatosis type 1	15 (3.5)	1.0

a Estimated population of the state of Bahia: 14,141,626 inhabitants. Observed prevalence expressed per 1,000,000 inhabitants.

Among the rare diseases with a confirmed diagnosis, 69.4% were included in the National Policy for Comprehensive Care of People with Rare Diseases (PNAIPDR), indicating that the diseases listed in the International Classification of Diseases (ICD) covered by the policy are incomplete, which highlights the need for expansion and updating. Moreover, due to the heterogeneous nature of this classification, many records that should have been allocated to the intellectual disabilities group were instead incorporated into the congenital anomalies group, as they lacked a corresponding category in the International Classification of Diseases (ICD). Thus, it is likely that the percentages obtained for the intellectual disabilities group are underestimated, as many cases may have been classified as congenital anomalies due to their syndromic forms and, therefore, would belong to the corresponding categories in the International Classification of Diseases (ICD).

This epidemiological survey was fundamental in providing a detailed understanding of the local reality, which will enable more effective management of resources and public health policies for individuals affected by rare diseases in this region. The study population was predominantly young, with the majority having a confirmed diagnosis, although experiencing a prolonged diagnostic odyssey; the principal rare diseases managed at the center were also identified. As a pioneering and comprehensive study, this work provided valuable insights into the landscape of rare diseases in Bahia.

## Data Availability

The data analyzed in this study are available in the REDCap software (https://projectredcap.org/), for which RARAS holds a license. Due to the General Data Protection Law (LGPD), Law No. 13,709/2018, access is restricted exclusively to researchers registered in the study. If necessary, the raw data can be made available through reports generated by REDcap. The author is available to clarify any questions or concerns.
