# Supplementary material for: Retrospective epidemiological study in a reference service for rare diseases, Salvador, 2018-2019
Source: Epidemiol Serv Saude. 2026 Apr 11;35:e20240832. doi: 10.1590/S2237-96222026v35e20240832.en (PMC13686371; doi:10.1590/S2237-96222026v35e20240832.en)
Supplement: Translation

# Translation: Estudo epidemiológico retrospectivo em serviço de referência em doenças raras, Salvador, 2018-2019

**DOI:** 10.1590/S2237-96222026v35e20240832.pt

**Authors:** Camila Ferreira Ramos, Ney Cristian Amaral Boa Sorte, Angelina Xavier Acosta

**Affiliations:** 1 Universidade Federal da Bahia, Programa de Pós-Graduação em Medicina e Saúde, Salvador, Bahia, Brasil Universidade Federal da Bahia Programa de Pós-Graduação em Medicina e Saúde Salvador Bahia Brasil; 2 Hospital Universitário Professor Edgard Santos, Núcleo de Avaliação em Tecnologias em Saúde, Salvador, BA, Brasil. Hospital Universitário Professor Edgard Santos Núcleo de Avaliação em Tecnologias em Saúde Salvador BA Brasil; 3 Universidade Federal da Bahia, Departamento de pediatria da Faculdade de Medicina da Bahia, Salvador, BA, Brasil. Universidade Federal da Bahia Departamento de pediatria da Faculdade de Medicina da Bahia Salvador BA Brasil

**Keywords:** Inquéritos Epidemiológicos, Doenças Raras, Genética, Serviços de Saúde, Hospitais Universitários

## Abstract

**Objetivo:**

Descrever um inquérito epidemiológico envolvendo indivíduos com doenças raras atendidos no período de 2018 a 2019 em um serviço de referência em doenças raras da Bahia.

**Métodos:**

Os dados foram obtidos a partir dos prontuários, sendo utilizados três formulários denominados como "identificação", "diagnóstico" e "tratamento", posteriormente inseridos no software REDcap e analisados de forma descritiva.

**Resultados:**

Foram incluídos 1.744 participantes, em que 69,3% pertenciam às faixas etárias de 0-19 anos (mediana de 15 anos), predominando o sexo feminino (51,1%). A maioria dos casos apresentava diagnóstico confirmado e os casos com suspeita diagnóstica e sem diagnóstico definido representavam 20,1% e 18,0%, respectivamente. A maioria dos diagnósticos se deu clinicamente, 58,4%. O tempo médio da idade do início dos sintomas foi 7,5 anos. A recorrência familiar foi relatada em 36,7% dos casos e a consanguinidade em 16,1%. A média da idade materna na concepção foi 26,6 anos e a idade paterna foi 31,0 anos. Cerca de 94,0% das doenças raras foram de origem genética, dentre essas, 69,4% estavam incluídas nas classificações internacionais de doenças da política nacional de atenção integral às doenças raras.

**Conclusão:**

Observou-se uma amostra predominantemente jovem, a maioria possuía diagnóstico confirmado, porém com atraso no diagnóstico, caracterizando, assim, o perfil sociodemográfico e clínico das doenças raras atendidas no centro, cujas informações têm sido relevantes para o planejamento de estratégias quanto à vigilância e à oferta de cuidados a essa população.

**Ethical aspects**

Esta pesquisa respeitou os princípios éticos, obtendo os seguintes dados de aprovação:

Comitê de ética em pesquisa: Universidade Federal da Bahia

Número do parecer: 5.961.780

Data de aprovação: 24/3/2023

Certificado de apresentação de apreciação ética: 33970820.0.3001.0049

Termo de consentimento livre e esclarecido: Dispensado.

## Introdução 

Doença rara é o termo usado para descrever condições geralmente crônicas e debilitantes, cuja incidência e/ou prevalência individual é baixa ^1^. Países como Austrália, Estados Unidos, Japão e Reino Unido adotam a prevalência de 1,1; 7,5; 4,0; 0,18 por 10 mil habitantes, respectivamente ^2^. No Brasil, o Ministério da Saúde define doença rara segundo o critério da organização mundial da saúde, ou seja, 1,3 a cada 2 mil indivíduos ou 65 a cada 100 mil indivíduos ^3^. Embora individualmente raras, em conjunto, elas afetam cerca de 10,0% da população. Estima-se a existência em torno de 6 mil condições, sendo 80,0% delas de origem genética ^4^, com prevalência mundial em torno de 3,5% a 5,9%, ou seja, cerca de 260-440 milhões de indivíduos. Extrapolando esses dados para o Brasil, estima-se que de 7 a 16 milhões de pessoas sejam acometidas com doenças raras, constituindo um impacto significativo na saúde pública ^5^. 

Dados epidemiológicos brasileiros acerca dessas doenças ainda são escassos, e muitos se baseiam em registros internacionais, especialmente patrocinados pela indústria farmacêutica e abordando grupos de doenças específicas. Esse aspecto restringe o conhecimento dos dados para gestores e formuladores de políticas públicas. Contudo, alguns avanços importantes ocorreram, com destaque para a criação da política nacional de atenção integral às pessoas com doenças raras no sistema único de saúde, instituída pela Portaria nº 199, de 30 de janeiro de 2014 ^3^. Essa política visa auxiliar a redução da morbimortalidade com a melhoria da qualidade de vida dos indivíduos através das ações de promoção, prevenção, detecção precoce, tratamento e cuidados paliativos. Nessa política, as doenças raras são classificadas em dois grandes grupos: as de etiologia genética, incluindo anomalias congênitas, deficiência intelectual e erros inatos do metabolismo; e as do grupo das doenças não genéticas (infecciosas, imunológicas e autoimunes) ^3^. 

Levando-se em consideração que a política nacional de atenção integral às pessoas com doenças raras, do Sistema Único de Saúde, não vinculou o processo diagnóstico e assistencial a um registro estruturado de dados, para além do preenchimento da autorização de procedimentos de alta complexidade, o estabelecimento do projeto "rede nacional de doenças raras", que está realizando um censo nacional a respeito das doenças raras com a descrição de aspectos clínicos, sociodemográficos e itinerários terapêuticos em 34 serviços de saúde do Brasil, constitui uma iniciativa fundamental para prover dados para a gestão pública. Na Bahia, o quarto maior estado em população do Brasil ^4^, baseado no dito previamente, estima-se algo entre 525 mil indivíduos e 885 mil indivíduos com doenças raras.

Nesse contexto, este trabalho objetiva descrever o panorama obtido pelo censo da rede nacional de doenças raras nos primeiros dois anos de execução dentro do maior serviço de referência em doenças raras da Bahia.

## Métodos 

Delineamento do estudo e contexto 

Tratou-se de estudo observacional multicêntrico, com delineamento transversal e com dados obtidos em 2022 e 2023, a partir do levantamento de casos com doenças raras atendidos em 2018 e 2019 nos ambulatórios da genética e da nutrologia e nutrição pediátrica do Hospital Universitário Professor Edgard Santos, da Universidade Federal da Bahia. 

A Bahia tem 564.760,429 km² de área, com uma população de 14.141.626 habitantes. Para a gestão da saúde, o estado está dividido em nove núcleos regionais de saúde, dentre os quais o foco recaiu especificamente sobre o Leste, no qual está a capital do estado, Salvador. 

Participantes/tamanho do estudo 

Foram incluídos todos os atendimentos realizados no centro em 2018 e 2019 que apresentaram diagnóstico confirmado, suspeita diagnóstica ou falta de diagnóstico definido de doença rara, com dispensa da aplicação do termo de consentimento livre e esclarecido e termo de assentimento livre e esclarecido, dado o caráter retrospectivo dos dados coletados. Dados incompletos não foram critérios de exclusão. Os casos incluídos foram obtidos a partir da busca de atendimentos registrados no prontuário eletrônico e físico do hospital. 

Fonte de dados 

Para a coleta de dados, por meio do aplicativo RedCAP, foram usados três formulários denominados como: "identificação", com dados sociodemográficos, "diagnóstico", com dados de formas e caracterização do diagnóstico, e "tratamento", com dados referentes à caracterização terapêutica dos casos.

Viés 

A equipe responsável pela coleta de dados foi previamente treinada pelos pesquisadores responsáveis do centro, a fim de diminuir riscos de vieses de informação.

Variáveis

Foram coletadas variáveis referentes a raça/cor da pele, região de moradia e nascimento, sexo, idade, condição diagnóstica da doença rara (confirmado/suspeita/sem diagnóstico), tipo de diagnóstico (etiológico ou clínico), consanguinidade (sim/não), recorrência familiar (sim/não), momento do diagnóstico (pré-natal, neonatal ou pós-natal), fonte pagadora do diagnóstico, idade do início dos sintomas, se realiza tratamento específico para doença rara, tipo de tratamento, fonte pagadora do tratamento e acompanhamento com outra especialidade. 

Métodos estatísticos 

Os dados foram apresentados com estatística descritiva, por meio de frequências simples, para variáveis categóricas, e medidas de tendência central e dispersão, para as quantitativas. As prevalências das doenças mais identificadas foram calculadas pela razão entre o total de doentes e a população média da Bahia, estimada em 2018 e 2019 e expressas para cada 1 milhão de habitantes. Os dados foram analisados no Microsoft Excel, versão Office 365.

## Resultados 

Foram incluídos no estudo 1.744 participantes, dos quais 51,1% eram do sexo feminino e 76,3% se autodeclararam pardos. A maioria desses indivíduos nasceu na região Nordeste do Brasil (97,2%), a maior parte (43,4%) do Núcleo Regional de Saúde Leste da Bahia, onde está localizado o centro estudado. A capital do estado, Salvador, teve a maior frequência de casos (34,0%). Quanto à idade dos participantes, a maioria (69,3%) estava entre 0-19 anos completos, com mediana de 15,0 (8,0-25,0) anos (Tabela 1). 

O núcleo regional de saúde de maior procedência de participantes foi o Leste (48,1%), com Salvador responsável por 71,8% dos casos, seguido do centro Leste, do Sul, do Centro Norte, do Sudoeste, do Nordeste, do Norte, do Oeste e do Extremo Sul (20,6%; 7,4%; 6,2%; 5,6%; 4,5%; 4,4%; 2,4%; 0,8%, respectivamente). 

Em relação à situação do diagnóstico, 1.084 participantes (61,9%) apresentavam diagnóstico confirmado, 352 (20,1%) participantes tinham suspeita diagnóstica e 315 (18,0%) participantes se mostraram sem diagnóstico definido de doença rara (Figura 1). A maioria dos diagnósticos foi definido por critérios clínicos (58,4%), e dentre os casos com diagnóstico etiológico (41,6%), o método bioquímico foi o que apresentou as maiores porcentagens (34,0%), seguido dos métodos citogenéticos (32,4%), molecular (17,9%) e anatomopatológico (34,0%). Além disso, sete indivíduos possuíam mais de um diagnóstico (1.751 registros oriundos de 1.744 indivíduos). 

**Tabela 1:** Características sociodemográficas do estudo epidemiológico retrospectivo. Salvador, 2018-2019 (n=1.744)

Variáveis	n (%)
Masculino	851 (48,8)
Feminino	892 (51,1)
Indefinido	1 (0,1)
Indefinido	1 (0,1)
Região de nascimento (núcleo regional de saúde) na Bahia	
Sudoeste	99 (5,7)
Sul	135 (7,7)
Oeste	44 (2,5)
Norte	74 (4,2)
Nordeste	63 (3,6)
Leste	755 (43,4)
Extremo Sul	14 (0,8)
Centro Norte	99 (5,7)
Centro Leste	290 (16,6)
Não informado	171 (9,8)
Faixa etária (anos)	
0-5	478 (27,4)
6-19	731 (41,9)
20-59	487 (27,9)
≥60	48 (2,8)

**Figura 1:** Status do diagnóstico do estudo epidemiológico retrospectivo. Salvador, 2018-2019 (n=1.751)

Na maioria dos casos, o diagnóstico ocorreu no período pós-natal 97,0%. O tempo médio da idade do início dos sintomas foi 7,5 (±12,8) anos e a mediana (p25-p75) foi 2,0 (1,0-7,0) anos. A recorrência familiar foi relatada em 36,7% dos casos e a consanguinidade observada em 16,1% dos casos. Sobre a idade materna no momento do nascimento do indivíduo com doença rara, a a média (desvio-padrão) foi 26,6 ^6^^,^^8^, variando de 13-47 anos. A idade paterna, por sua vez, mostrou uma média (desvio-padrão) de 31,0 ^8^^,^^3^, com variação anos (Tabela 2).

Dentre os casos com diagnóstico confirmado, as doenças mais prevalentes no centro estudado estão descritas na Tabela 3. Ao realizar uma análise mais detalhada sobre a classificação das doenças raras com diagnóstico confirmado, observou-se que 94,0% delas foram de origem genética, das quais 69,4% estavam incluídas na lista de doenças, codificadas pela classificação internacional de doenças e contempladas na política nacional de atenção integral às pessoas com doenças raras. Dentre essas doenças listadas, 74,1% faziam parte do grupo das anomalias congênitas, 22,6% eram do grupo dos erros inatos do metabolismo e 3,3% classificadas no grupo de deficiência intelectual. Referente às doenças raras de origem não genética, 7,8% se enquadram como doenças infecciosas, 14,1% como de causa inflamatória, 23,4% como autoimunes e 54,7% como outras doenças raras de causa não genética. 

Como nem todas as doenças raras diagnosticadas estavam presentes na lista da política nacional de atenção integral às pessoas com doenças raras, dentre as doenças codificadas pela classificação internacional de doenças que não estavam incluídas na lista, 43,0% pertenciam ao grupo das anomalias congênitas, 38,0% eram classificadas como erros inatos do metabolismo e 19,0% como deficiência intelectual (Figura 2). 

**Tabela 2:** Características clínicas do estudo epidemiológico retrospectivo. Salvador, 2018-2019 (n=1.751)

Variáveis	n (%)
Período do diagnóstico	
Pré-natal	11 (1,0)
Triagem neonatal	22 (2,0)
Pós-natal	1.051 (97,0)
Total	1.084 (100,0)
Recorrência familiar	
Sim	524 (36,7)
Não	896 (63,3)
Subtotal	1.420 (100,0)
Não informado	331 (18,9)
Consanguinidade	
Sim	193 (16,1)
Não	1.002 (83,9)
Subtotal	1.195 (100,0)
Não informado	556 (31,7)

**Figura 2:** Distribuição das doenças raras de origem genética e não genética do estudo epidemiológico retrospectivo do Hospital Universitário Professor Edgard Santos. Salvador, 2018-2019 (n=1.084)

## Discussão 

Os estudos acerca da epidemiologia das doenças raras no Brasil são escassos, dessa forma este trabalho visou contribuir com informações abrangentes sobre indivíduos com doenças raras no serviço de referência em doenças raras do Hospital Universitário Professor Edgard Santos, para o melhor conhecimento dessa população em um centro de grande representação na Bahia. 

Foram identificadas algumas limitações operacionais neste estudo referentes à fonte de dados, já que, por se tratar de uma revisão de prontuários, a falta da padronização de evoluções médicas gerou algumas lacunas no preenchimento das informações. Entretanto, não houve grande comprometimento, uma vez que foram utilizadas estratégias para obter a completude dos dados através da leitura detalhada das informações disponibilizadas nas evoluções médicas a partir de uma padronização da coleta de dados por uma equipe treinada e supervisionada constantemente pela coordenação da pesquisa.

Os dados sociodemográficos deste trabalho refletiram o último censo demográfico do Instituto Brasileiro de Geografia e Estatística (IBGE) para a Bahia, em que 51,7% da população baiana é composta por mulheres, sendo os percentuais do estudo de 51,1% participantes desse sexo. Achados similares foram encontrados em um estudo em nível nacional, em que 50,6% da amostra era do sexo feminino ^6^. 

Referente à raça/cor da pele, a mais prevalente foi a parda (66,7%), seguida da preta (10,6%). Segundo o último censo de 2022 do IBGE para a Bahia, a raça/cor da pele parda apresentou percentuais inferiores (57,3%), e a raça/cor da pele preta apresentou maior frequência (22,4%) ^7^. O estudo em nível nacional apresentou a raça/cor da pele parda como a mais frequente (47,4%), seguida da branca (45,9%), muito provavelmente pela participação de indivíduos das regiões Sul e Sudeste, em que esse contingente é predominantemente de raça/cor da pele branca ^6^.

A maioria da amostra estudada (61,8%) encontrava-se na faixa etária de 0-19 anos completos; 20,1% dos indivíduos possuíam até 6 anos de idade. Em um estudo, 69,9% da casuística fazia parte do grupo pediátrico, corroborando a maior prevalência dos casos de doenças raras nos grupos da infância e da adolescência, por se tratar de doenças graves com manifestação precoce e detecção nessa faixa etária ^8^.

Referente à região de moradia, a região Leste do Núcleo Regional de Saúde da Bahia apresentou a maior prevalência (48,1%) de doenças raras, por abrigar Salvador, onde fica localizado o centro estudado e onde há o maior número de habitantes do estado ^7^. Extrapolando os dados para os indivíduos com doenças raras em relação ao número total de habitantes em cada região, observou-se que 0,017% dos indivíduos residentes na região Leste eram acometidos com doenças raras, seguido das regiões Centro Leste, 0,01% indivíduos, e Sul, 0,007% indivíduos. Para a observação de menores percentuais de participantes em regiões distantes da capital, supõe-se a dificuldade de acesso ou estarem sendo acompanhados em centros mais próximos, incluindo de outros estados, ou ainda que a prevalência seja reduzida e, por isso, haja menor representatividade, sendo necessário conhecer melhor a prevalência nas áreas mais remotas. 

Apesar de a maioria dos indivíduos possuir diagnóstico confirmado (61,9%), uma parcela significativa de indivíduos ainda configurava como diagnóstico suspeito (20,1%) ou sem diagnóstico definido (18,0%). Essas taxas elevadas de casos sem definição diagnóstica podem ser explicadas pelo acesso limitado às técnicas de diagnóstico molecular à época da coleta deste estudo, realidade semelhante a de outros serviços públicos no Brasil ^9^. Os dados em nível nacional mostraram que os casos ainda configurados como suspeita diagnóstica representavam 17,0% da amostra ^6^. Considerando a complexidade das doenças raras, o diagnóstico pode ser desafiador até mesmo em países desenvolvidos, como Estados Unidos e Austrália, os quais dispõem de serviços especializados e com melhor acesso aos exames genéticos, apresentando, porém uma quantidade expressiva de casos sem diagnóstico ^10^. Antes da implementação da política, estudos em nível nacional mostraram que os testes moleculares estavam presentes em 50,0% dos serviços genéticos listados no Sistema Único de Saúde ^11^. Um estudo realizado sobre a disponibilidade dos testes genéticos em serviços públicos no Brasil apresentou dados similares aos estudos anteriores, com uma presença de 54,1% ^9^.

A maioria (58,4%) dos casos com diagnóstico confirmado foi diagnosticado de forma clínica, e 41,6% tiveram etiologia definida, sendo semelhante aos dados em nível nacional. Quanto ao tipo de diagnóstico, o bioquímico apresentou os maiores percentuais (34,0%), semelhante ao estudo nacional, que foi 42,5% ^6^. Referente ao diagnóstico molecular, o centro estudado apresentou percentuais de confirmação diagnóstica (17,9%) quando comparado ao observado no Brasil (30,9%). Essa diferença foi quase duas vezes menor, o que pode ser explicado pelo fato de as regiões Sul e Sudeste apresentarem maiores disponibilidades de testes moleculares, devido aos serviços possuírem laboratórios mais bem equipados e com maior disponibilidade de exames laboratoriais quando comparados aos demais estados do Brasil ^6^. A maior disponibilidade desses testes certamente possibilita um diagnóstico mais assertivo, minimizando o impacto da jornada diagnóstica ^12^.

A taxa de consanguinidade foi 16,1%, sendo bem superior aos dados nacionais (6,4%), condizente com estudos prévios na região Nordeste do país, que exibem taxas mais altas de consanguinidade e de endogamia, com uma variação de 6,0% a 12,0% ^13^. Um estudo realizado no Rio Grande do Norte mostrou que os municípios com maior isolamento geográfico apresentaram maiores percentuais de indivíduos aparentados (30,0%) quando comparados aos locais com maior mobilidade (9,0%). Por outro lado, estudos anteriores indicaram taxas menores de indivíduos aparentados na América do Sul (0,96%) e no Brasil (1,60%) ^14^. A maioria (52,1%) dos indivíduos consanguíneos residiam em municípios localizados no interior do estado, muitos em áreas rurais, em que as práticas de casamento consanguíneos são mais frequentes, por diversas razões, como questões culturais e de isolamento geográfico ^13^. 

Outro fator de risco relacionado com a ocorrência de doenças genéticas é a idade dos pais na concepção. Porém, a média das idades materna e paterna observada foi 26 anos e 31 anos, respectivamente, inferior à relacionada na literatura ^15^^-^^16^. Assim, a priori, esse fator de risco não se mostrou relevante, porém faz-se necessária uma análise mais detalhada para se conhecer a vulnerabilidade da população estudada e, com isso, contribuir em melhores estratégias de prevenção.

Em relação à idade do início dos sintomas, foi de 7,5 anos, sendo similar ao encontrado no estudo nacional, cuja média foi 5,4 anos ^6^. Esse atraso pode ser ainda maior para os países em desenvolvimento, como o Brasil ^17^, cuja jornada diagnóstica pode ser um fator determinante no aumento dos encargos financeiros relacionados ao diagnóstico e/ou ao tratamento, além de agravamento na qualidade de vida aos acometidos com doenças raras e seus familiares ^18^.

Dentre as doenças raras diagnosticadas, observou-se que as dez mais prevalentes corresponderam a 39,7% da casuística estudada, e os demais diagnósticos (60,3%) correspondiam a doenças com representatividade individual de muita baixa frequência. Esses resultados estão condizentes com a base de dados orphanet, em que a maioria (84,5%) tem prevalência menor, em contraste com um pequeno grupo de doenças raras com intervalos de prevalência mais comum ^18^. Nota-se que as dez doenças mais prevalentes (Tabela 3) pertenciam ao grupo das anomalias congênitas, segundo a Política Nacional de Atenção Integral às Pessoas com Doenças Raras, sendo a osteogênese imperfeita a mais prevalente (9,6: 1 milhão de habitantes). No Brasil, a prevalência para essa doença ainda não é clara, pois são escassos os dados sobre o total de casos diagnosticados em plataformas oficiais ^19^. Estima-se que a maioria dos indivíduos acometidos com osteogênese imperfeita na Bahia seja acompanhada no centro estudado, pois é o único serviço público habilitado no estado para o tratamento dessa doença ^20^. Essa maior prevalência contribuiu com 37,8% da casuística observada em nível nacional, em que a osteogênese imperfeita foi a quarta mais frequente. Esse resultado tem que ser interpretado com muito cuidado, pois existe um claro viés de seleção nos dados. O centro analisado é referência para muitas das doenças raras mais prevalentes descritas, o que justifica essa distribuição.

**Tabela 3:** Doenças raras mais prevalentes do estudo epidemiológico retrospectivo. Salvador, 2018-2019 (n=431)

Doenças raras mais prevalentes	n (%)	Prevalência observada^a^
Osteogênese imperfeita	136 (31,5)	9,6
Síndrome de turner	58 (13,5)	4,1
Distrofia muscular	56 (13,0)	3,9
Transtornos adrenogenitais	47 (10,9)	3,1
Mucopolissacaridose	40 (9,3)	2,8
Fibrose cística	22 (5,1)	1,6
Angioedema hereditário	22 (5,1)	1,6
Epidermólise bolhosa	19 (4,4)	1,3
Glicogenose	16 (3,7)	1,1
Neurofibromatose tipo I	15 (3,5)	1,0

a População estimada da Bahia: 14.141.626 habitantes. Prevalência observada expressa para cada 1 milhão de habitantes.

Neste estudo, 94,0% das doenças raras eram de origem genética (Figura 1), um percentual maior do que os achados descritos na literatura, em que cerca de 80,0% das doenças raras são de origem genética ^21^. Apesar das estratégias empregadas para minimizar o viés de seleção, como descrito na metodologia, esse percentual foi influenciado pelo fato de que a maioria da casuística foi proveniente de pacientes acompanhados em ambulatórios de genética. Supõe-se que as doenças raras de origem não genética estejam subnotificadas pelas razões metodológicas já explicadas. 

Dentre as doenças raras com diagnóstico confirmado, 69,4% delas estavam incluídas na Política Nacional de Atenção Integral às Pessoas com Doenças Raras, demonstrando claramente que as doenças descritas na classificação internacional de doenças previstas na política encontram-se incompletas, havendo necessidade de ampliação e de atualização. Além disso, com o fato de essa classificação ser heterogênea, muitos registros que deveriam estar alocados no grupo das deficiências intelectuais, foram incorporados ao grupo das anomalias congênitas por não possuir uma classificação internacional de doenças prevista. Assim, é provável que os percentuais obtidos referente ao grupo das deficiências intelectuais estejam subestimados, muitos deles podem ter sido incluídos como anomalias congênitas por se tratar de formas sindrômicas e, então, pertencerem a classificações internacionais de doenças previstas nesse grupo.

Este inquérito epidemiológico foi fundamental para conhecer de maneira detalhada a realidade local, o que possibilitará esforços para uma melhor gestão dos recursos e das políticas de saúde pública aos acometidos com doenças raras nessa região. Observou-se uma amostra predominantemente jovem, em que a maioria possuía diagnóstico confirmado, porém com uma odisseia diagnóstica longa, sendo identificadas as principais doenças raras atendidas no centro. Por se tratar de um estudo pioneiro e abrangente, este trabalho contribuiu com informações valiosas sobre o cenário das doenças raras na Bahia.

## Data Availability

Os dados analisados neste estudo estão disponíveis no SOFTWARE REDcap (https://projectredcap.org/), onde a RARAS possui uma licença junto com o REDcap. Por conta da Lei Geral de Proteção de Dados Pessoais (LGPD), Lei nº 13.709/2018, o acesso é restrito apenas aos pesquisadores cadastrados na pesquisa. Se necessário, disponibilizamos os dados brutos através dos relatórios gerados do REDcap. O autor está disponível para elucidar quaisquer tipos de dúvidas.
